# A Geographically-Restricted but Prevalent *Mycobacterium tuberculosis* Strain Identified in the West Midlands Region of the UK between 1995 and 2008

**DOI:** 10.1371/journal.pone.0017930

**Published:** 2011-03-25

**Authors:** Jason T. Evans, Robert L. Serafino Wani, Laura Anderson, Andrea L. Gibson, E. Grace Smith, Annette Wood, Babatunde Olowokure, Ibrahim Abubakar, Jonathan S. Mann, Sarah Gardiner, Helen Jones, Pam Sonnenberg, Peter M. Hawkey

**Affiliations:** 1 Health Protection Agency West Midlands Laboratory, Heart of England NHS Foundation Trust, Bordesley Green East, Birmingham, United Kingdom; 2 Department of Infection, Guy's and St Thomas' NHS Foundation Trust, London, United Kingdom; 3 Respiratory Diseases Department - Tuberculosis Section, Health Protection Agency Centre for Infections, London, United Kingdom; 4 Health Protection Agency West Midlands East Health Protection Unit, Birmingham, United Kingdom; 5 Health Protection Agency West Midlands Regional Epidemiology Unit, Birmingham, United Kingdom; 6 Department of Respiratory Medicine, New Cross Hospital, Wolverhampton, United Kingdom; 7 Department of Microbiology, New Cross Hospital, Wolverhampton, United Kingdom; 8 Department of Infection & Population Health, Centre for Sexual Health & HIV Research, University College London, London, United Kingdom; 9 School of Immunity and Infection, College of Medical and Dental Sciences, University of Birmingham, Birmingham, United Kingdom; Fundació Institut Germans Trias i Pujol; Universitat Autònoma de Barcelona CibeRES, Spain

## Abstract

**Background:**

We describe the identification of, and risk factors for, the single most prevalent *Mycobacterium tuberculosis* strain in the West Midlands region of the UK.

**Methodology/Principal Findings:**

Prospective 15-locus MIRU-VNTR genotyping of all *M. tuberculosis* isolates in the West Midlands between 2004 and 2008 was undertaken. Two retrospective epidemiological investigations were also undertaken using univariable and multivariable logistic regression analysis. The first study of all TB patients in the West Midlands between 2004 and 2008 identified a single prevalent strain in each of the study years (total 155/3,056 (5%) isolates). This prevalent MIRU-VNTR profile (32333 2432515314 434443183) remained clustered after typing with an additional 9-loci MIRU-VNTR and spoligotyping. The majority of these patients (122/155, 79%) resided in three major cities located within a 40 km radius. From the apparent geographical restriction, we have named this the “Mercian” strain. A multivariate analysis of all TB patients in the West Midlands identified that infection with a Mercian strain was significantly associated with being UK-born (OR = 9.03, 95%CI = 4.56–17.87, p<0.01), Black Caribbean (OR = 5.68, 95%CI = 2.96–10.91, p<0.01) resident in Wolverhampton (OR = 9.29, 95%CI = 5.69–15.19, p<0.01) and negatively associated with age >65 years old (OR = 0.25, 95%CI = 0.09–0.67, p<0.01). A second more detailed investigation analyzed a cohort of 82 patients resident in Wolverhampton between 2003 and 2006. A significant association with being born in the UK remained after a multivariate analysis (OR = 9.68, 95%CI = 2.00–46.78, p<0.01) and excess alcohol intake and cannabis use (OR = 6.26, 95%CI = 1.45–27.02, p = .01) were observed as social risk factors for infection.

**Conclusions/Significance:**

The continued consistent presence of the Mercian strain suggests ongoing community transmission. Whilst significant associations have been found, there may be other common risk factors yet to be identified. Future investigations should focus on targeting the relevant risk groups and elucidating the biological factors that mediate continued transmission of this strain.

## Introduction

DNA fingerprinting of *Mycobacterium tuberculosis* has a key role in TB control and cluster investigation as the molecular data obtained can be used to direct and focus public health control efforts [1], [2]. For example, DNA fingerprinting enhanced the investigation of a large outbreak in North London where many of the epidemiological links would not have been established by routine contact tracing or traditional epidemiological investigations alone [3]. Large-scale studies of *M. tuberculosis* strains have also enabled the assessment of the impact of global strain migration and the transmission dynamics of specific strains on a local or regional level [4]–[8].

The number of cases of tuberculosis in the UK has consistently increased each year since the late 1980s with 8,655 cases (14.1 cases per 100,000 population) diagnosed in 2008. There were 1,012 clinical cases (18.7 per 100,000) in the West Midlands region of the UK with a 43% increase in case numbers in the region since 2000. Birmingham is the largest city in the West Midlands with a rate of 42.4 cases per 100,000 in 2008. There were 44.3 cases per 100,000 in London in 2008. There is large variation in the incidence of TB across the West Midlands, with rates highest in one urban area of Birmingham (>80 cases per 100,000) and lowest in rural Worcestershire (<4 cases per 100,000 in 2008) [9].

We analyzed all *M. tuberculosis* isolates in the West Midlands region of the UK from 2004 and 2008 by universal prospective DNA fingerprinting and identified the most prevalent strain. We then examined the geographical distribution and epidemiological characteristics of cases infected with this strain in the West Midlands region, and in the city of Wolverhampton, which was found to have the highest proportion of patients with this strain.

## Methods

### Study Population

The setting for this study was the West Midlands region of the UK. This region had a total population of 5.4 million in 2008. The city of Birmingham has the largest population in the West Midlands with one million inhabitants [10]. Prospective universal DNA fingerprinting was undertaken between 2004 and 2008 with retrospective genotyping carried out on strains isolated before 2004. Retrospective observational epidemiological investigations were undertaken within one city and on a regional scale.

### Case definition

Patients with the MIRU-VNTR profile of the most prevalent *M. tuberculosis* strain in the West Midlands were included in further epidemiological investigation.

### Mycobacterial strains and DNA fingerprinting

The HPA Midlands Regional Centre for Mycobacteriology undertook IS*6110* Restriction Fragment Length Polymorphism (RFLP) typing on specific requested *M. tuberculosis* strains until 2004. From 2004 onwards, all *M. tuberculosis* isolates received from laboratories in the West and East Midlands at the HPA Midlands Regional Centre for Mycobacteriology at Birmingham Heartlands Hospital identified as members of the *M. tuberculosis* complex were analysed by MIRU-VNTR (Mycobacterial Interspersed Repetitive Units containing Variable Number Tandem Repeats) typing using 15 loci as previously described (ETR-A, -B, -C, -D, -E and MIRU-02, -10, -16, -20, -23, -24, -26, -27, -39, -40) [1], [11], [12]. The most recent complete calendar year available for molecular and epidemiological data analysis was 2008. A selection of strains were analysed by nine additional loci (Mtub04, Mtub21, QUB-11b, Mtub29, Mtub30, Mtub34, Mtub39, QUB-26, QUB-4156) that together with the 15 loci comprise the internationally optimised set of 24 loci [13].

### *M. tuberculosis* strain clustering analysis

MIRU-VNTR and IS*6110* RFLP data were entered into BioNumerics v5.1 (Applied Maths, Saint Marten-Latem, Belgium) to identify clustered MIRU-VNTR profiles. MIRU-VNTR data was queried against the HPA UK *Mycobacterium tuberculosis* Strain Typing Database which contains 26,879 typed *M. tuberculosis* strains from six contributing centres in the United Kingdom [14].

### Confirmation of the most prevalent MIRU-VNTR profile as a single strain by IS*6110* RFLP

For *M. tuberculosis* isolates originally cultured between 1995 and 2003, DNA fingerprinting was undertaken retrospectively on requested clusters with significant epidemiological links. Strains were retrospectively analyzed by MIRU-VNTR and IS*6110* RFLP typing to confirm the genetic relatedness within clusters. IS*6110* RFLP interrogates a different and independent genetic sequence than MIRU-VNTR typing. IS*6110* RFLP was undertaken in accordance with the international standardized protocol using *M. tuberculosis* strain MT14323 as a control strain [15].

### Assignation of global clade lineage

Spoligotyping was carried out to identify the global strain family that the most prevalent strain is part of. Spoligotyping was performed using the Luminex Multianalyte Profiling System as previously described [16]. Spoligotype families were assigned by comparison to the international SpolDB4 database [17].

### West Midlands regional epidemiological analysis

Patients with *M. tuberculosis* strains identified as the Mercian strain between 2004 and 2008 were compared to all patients with tuberculosis in the West Midlands between 2004 and 2008 in the HPA Enhanced Tuberculosis Surveillance System. The HPA Enhanced Tuberculosis Surveillance System contains molecular, pathological, and treatment data on all notified cases of tuberculosis in England including culture-confirmed cases and clinically diagnosed cases. All patients with strain typing data were selected for comparison. Two levels of patient residence were analyzed: West Midlands Health Protection Unit and Local Authority. Local HPA Health Protection Units work alongside the National Health Service in England providing specialist support in communicable disease and infection control. There are three Health Protection Units in the West Midlands: West Midlands East, West Midlands North, and West Midlands West. The major cities of Birmingham and Coventry are located in West Midlands East, Stoke-on-Trent in West Midlands North, and Wolverhampton in West Midlands West. More specific analysis of patient residence was undertaken by analyzing patient location within one of 33 Local Authority regions in the West Midlands. Local Authorities are administrative regions that are based on city or county boundaries.

### Geographical distribution of the Mercian strain within the West Midlands region

Laboratory records of patients with the most prevalent strain were used to map patient residential location using postcode within the West Midlands.

### City specific epidemiological investigation

When it became apparent that there was a cohort of patients in Wolverhampton with an indistinguishable MIRU-VNTR profile, a retrospective review of patient case notes and interview of specialist tuberculosis nurses who were involved with the care of these patients was undertaken for culture-positive patients resident in Wolverhampton diagnosed with the same indistinguishable MIRU-VNTR profile between June 2003 and February 2006 to identify common factors and potential epidemiological links. These patients were compared to culture-positive cases diagnosed with other strains in 2004.

A questionnaire was designed to collect comprehensive epidemiologic information including demographic characteristics, clinical history, predisposing risk factors and evidence of contact with patients with active disease caused by any strain. Information was also obtained on occupational, social and recreational history, compliance with tuberculosis treatment and change in weight after eight weeks treatment. Chest radiographs of all patients were reviewed for the presence of cavitation.

### Statistical analysis

Proportions calculated from epidemiological data obtained from the West Midlands regional and Wolverhampton city datasets were compared using Pearson's chi-squared test with Fisher's exact test where necessary. Univariate and multivariate logistic regression modeling was used to test the significance of odds ratios in Stata v10 (Stata Corp, College Station, TX, USA). The multivariable model was assembled by adding covariates individually in decreasing order of significance and the “goodness of fit” of each model was assessed using the likelihood ratio test. All cases with missing values for the variables examined were excluded from the multivariate model with 114 patients infected by the Mercian strain and 1,891 patients in the control group included. Differences in proportions between entries with complete data for each variable and missing data for at least one variable was analysed. A univariate analysis of the epidemiological investigation of patients resident in Wolverhampton was undertaken using EpiData Analysis v2.2 (EpiData Association, Odense, Denmark). The extent of any association was expressed as an odds ratio (OR) with 95% confidence intervals.

### Ethics Statement

This report details the current status of the investigation into the most prevalent strain in the West Midlands, which has been undertaken as part of normal public health practice by microbiologists, respiratory physicians, and public health teams. Therefore, specific ethical approval was not required. The Health Protection Agency (HPA) has Patient Information Advisory Group permission under the Health and Social Care Act 2001 to collect and analyse such data for public health purposes.

## Results

### *M. tuberculosis* strains

**Figure 1:**
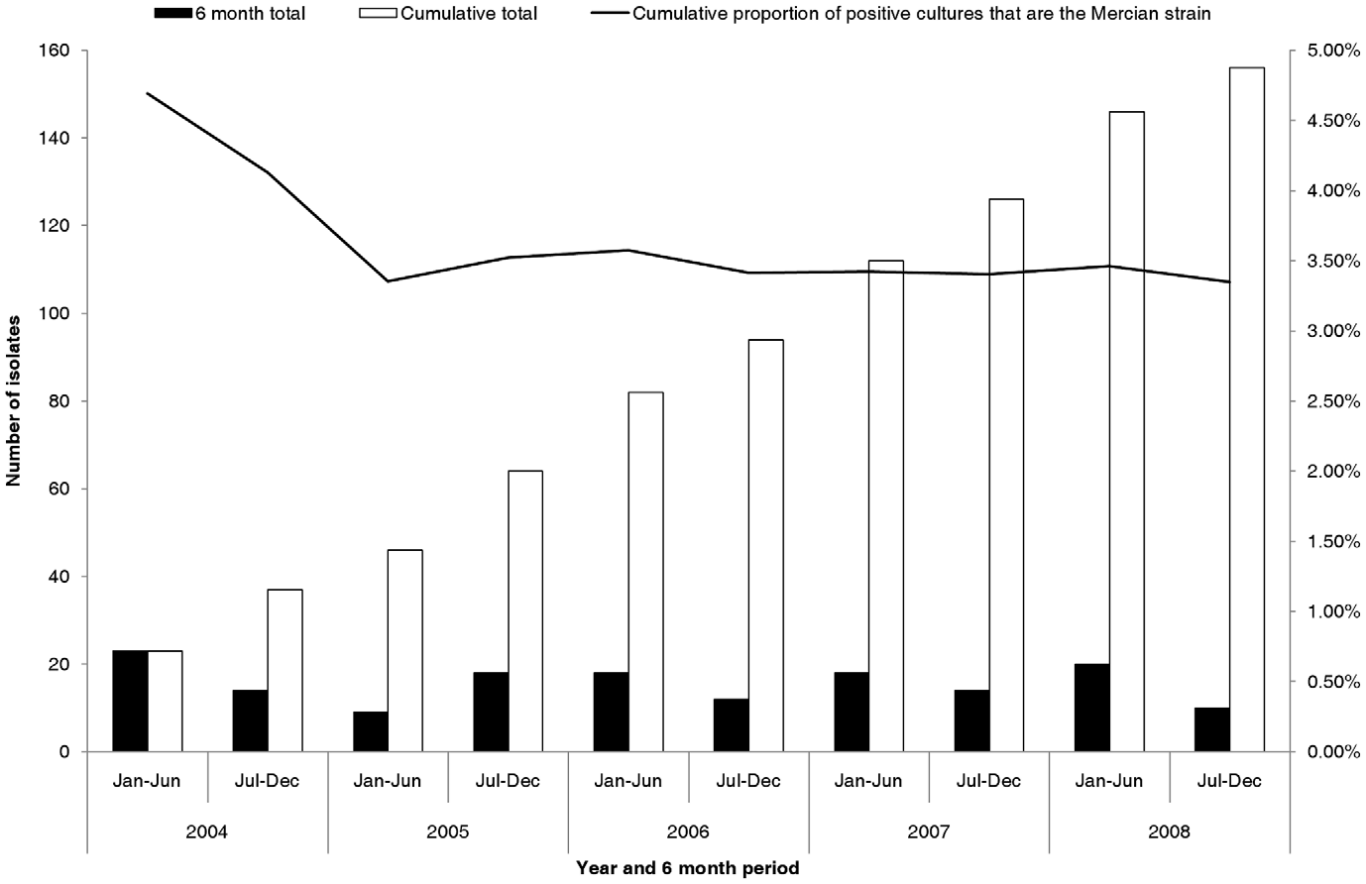
Incidence and cumulative total of the number of patients with the Mercian strain between 2004 and 2008.

Between 2004 and 2008, 4,830 isolates were typed from 31 referring laboratories in the West and East Midlands. Duplicate isolates (n = 171) were removed so that 4,659 isolates representing the first typed isolate from each patient between 1^st^January 2004 and 31^st^ December 2008 were selected. From 4,659 isolates typed, 3,056/4,659 (66%) originated from patients resident in the West Midlands and 1,603/4,659 (34%) from patients resident in the East Midlands. The single most prevalent MIRU-VNTR profile was 32333 2432515314, which was identified in 156/4,659 (3%) isolates across the West and East Midlands and 155/3,056 (5%) isolates in the West Midlands. Only one strain with this MIRU-VNTR profile was identified in the East Midlands. This profile was identified soon after universal prospective DNA fingerprinting was initiated in 2004 and has been consistently identified since then (Figure 1). The second and third most prevalent profiles were 42235 2542517333 (117/4,659, 3%) and 42235 2642515333 (88/4,659, 2%).

### Case definition

Inclusion of a patient for further epidemiological investigation was based on MIRU-VNTR typing, with a confirmed case defined as a patient with microbiologically confirmed tuberculosis and an isolate that had the 32333 2432515314 MIRU-VNTR profile.

### UK distribution of the most prevalent MIRU-VNTR profile in the West Midlands

The HPA UK *Mycobacterium tuberculosis* Strain Typing Database was interrogated to analyze the national distribution of the most prevalent strain in the West Midlands with a total of 176 isolates identified across the UK between 2004 and 2008. Only 6/162 (4%) of these isolates were identified in patients resident outside of the Midlands. Since this MIRU-VNTR profile appeared to be geographically restricted to the West Midlands in the UK, we have named the profile the “Mercian strain”, after the Anglo-Saxon kingdom of Mercia [18].

### Geographical distribution of the Mercian strain within the West Midlands region

**Figure 2:**
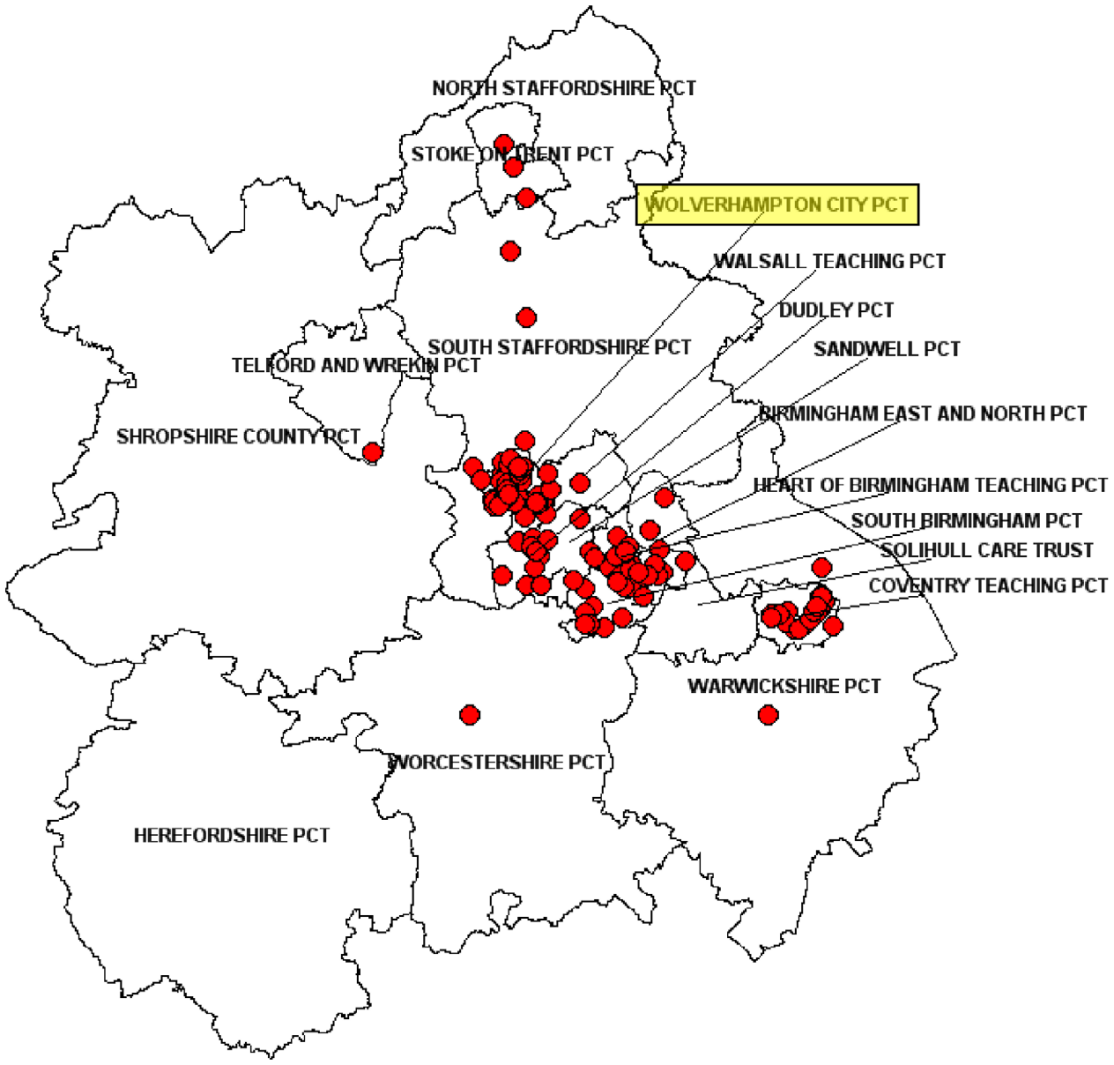
Geographical mapping of cases with the Mercian strain in the West Midlands between 2004 and 2008. Primary Care Trusts (PCTs) are regional NHS organizations in England that provide primary and community care. The city of Wolverhampton where the focused epidemiological investigation was undertaken is highlighted in yellow. The postcode map was produced under license by ©Crown Copyright and database right 2010. Ordnance Survey License 100016969/100022432.

Three major cities in the West Midlands were the focus for this strain as 55/156 (35%) isolates originated from Wolverhampton, 41/156 (26%) isolates from Birmingham, and 26/156 (17%) isolates from Coventry. Overall, 122/156 (78%) Mercian isolates were identified in patients resident in one of these three cities which are located within a 40 km radius of each other. The Mercian strain accounted for 21% (53/258) of all *M. tuberculosis* isolates in Wolverhampton compared to 9% (27/285) of all strains in Coventry and 3% (41/1,243) in Birmingham. Figure 2 shows the geographical mapping of 32333 2432515314 in the West Midlands between 2004 and 2008.

### Confirmation of the 32333 2432515314 MIRU-VNTR profile as a distinct strain

**Figure 3:**
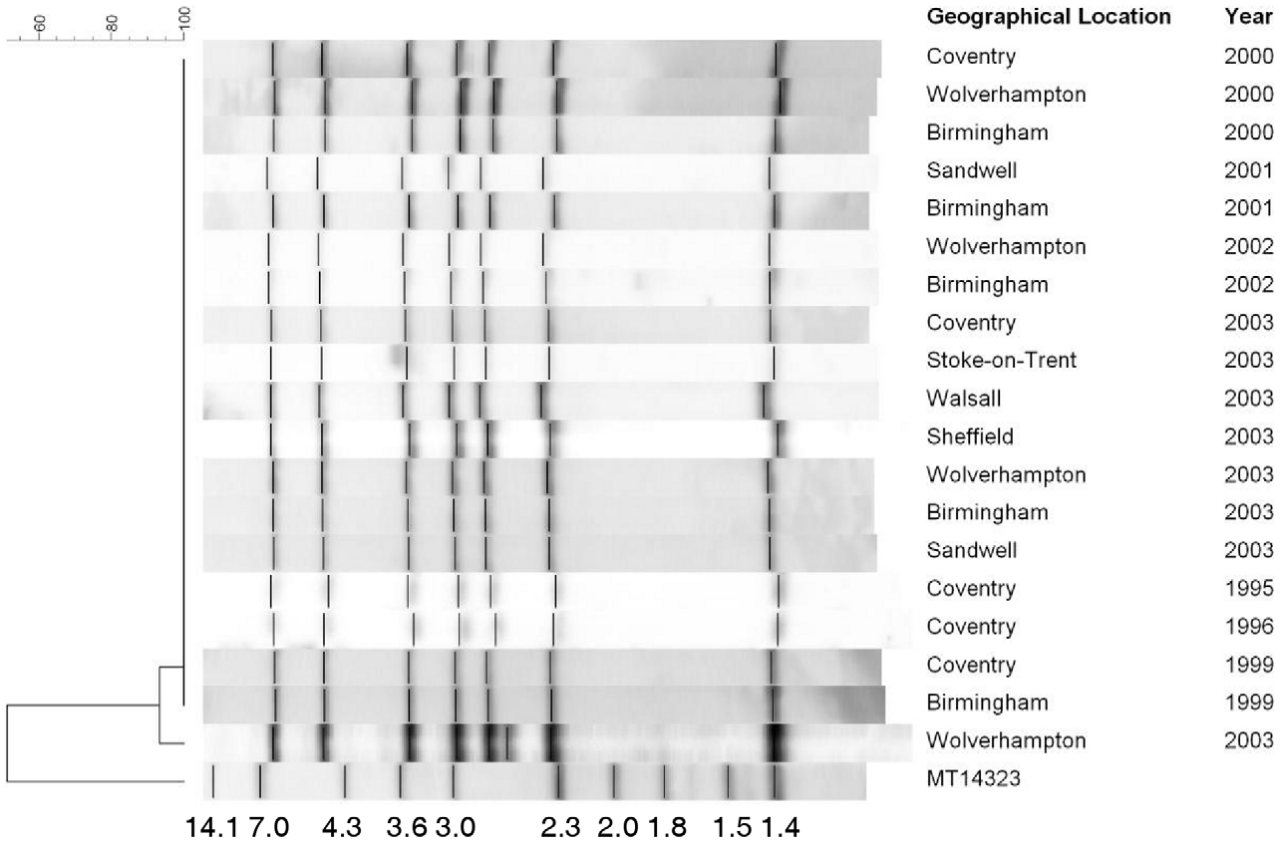
Results of RFLP analysis for isolates of the Mercian strain across the Midlands between 1995 and 2003. Each horizontal lane is an example of an individual *M. tuberculosis* strain from each location and year. The molecular sizes of the digested DNA fragments of MT14323 are shown in kilobases. The 8^th^ IS*6110* fragment was identified in an isolate from Wolverhampton in 2003.

Retrospective typing of stored *M. tuberculosis* isolates resulted in the identification of an additional 51 isolates from six different locations as members of the 32333 2432515314 Mercian strain MIRU-VNTR profile. Upon further investigation by IS*6110* RFLP, 50/51 (98%) of these strains were still indistinguishable (Figure 3) with a 7-band RFLP pattern. One isolate from Wolverhampton in 2003 possessed 8 copies of IS*6110* but this can still be considered as part of the same strain and the MIRU-VNTR profile did not vary in this single isolate. A selection of 10 strains from five different locations isolated between 2004 and 2008 were analyzed by the optimal 24 MIRU-VNTR loci set and spoligotyping. All 10 strains were indistinguishable at each of the 24 loci (32333 2432515314 434443183) and possessed an indistinguishable spoligotype (octal type 767776777760771) which is shared type (ST) 490 and is a member of the Clade X1 global clade. Investigation of the global SpolDB4 database revealed that only 3 strains with this shared type have been identified. The 3 other strains that are members of ST490 were identified as single strains in London, New York and Washington, DC.

### West Midlands regional epidemiological analysis

A total of 124/156 (79%) tuberculosis patients with the Mercian strain were successfully matched to notification data in the HPA Enhanced Tuberculosis Surveillance system. There were 2,066 tuberculosis patients with other strain types notified in the West Midlands between 2004 and 2008. Patient characteristics identified as risk factors significant in a univariate analysis (Table 1 and Table S1 for all epidemiological variables) were residence in the West Midlands West Health Protection Unit Area and then specifically residence in Wolverhampton, UK-born, and Black Caribbean or White ethnic group. Significant negative associations were identified with age not greater than 65 years old, the Black African ethnic group or extra-pulmonary disease. No significant association with resistance to any of the 1^st^ line tuberculosis drugs (p = .79) or multi-drug resistance (p = .92) was identified. The significant variables were then included in a multi-variate logistic regression which identified that being UK-born, Black Caribbean ethnic group, >65 years old, and resident in Wolverhampton, were significantly associated with the Mercian strain (Table 1). Age >65 years old was a significantly negative association with the Mercian strain. Therefore, age <65 years old is positively associated with the Mercian strain. There were no statistically significant differences between patients with complete data for each variable compared to those with missing data in both this analysis and the following city-wide epidemiological investigation.

**Table 1:** Univariate and multi-variate analysis of sociodemographic, clinical, and bacteriological data for patients with the Mercian strain (n = 124) and all other patients with strain typing data (n = 2,066) in the West Midlands between 2004 and 2008.

	No. patients		Unadjusted			Adjusted		
Variable	Mercian(n = 124)	WT(n = 2,066)	Odds Ratio	95% CI	P	Odds Ratio	95% CI	p
Gender								
Male	77	1,118	1.38	0.95–2.01	0.09	1.03	0.65–1.62	0.91
Age group (years)								
0–14	4	45	1.27	0.45–3.59	0.69	0.35	0.07–1.63	0.18
15–44	92	1310	1.00	1.00	Reference			
45–64	23	379	0.86	0.54–1.38	0.543	0.77	0.42–1.41	0.40
>65	5	330	0.22	0.09–0.53	<0.01*	0.25	0.09–0.67	<0.01*
HPU location in the West Midlands								
East	47	1,126	1.00	1.00	Reference			
North	10	206	1.16	0.58–2.34	0.67			
West	67	727	2.21	1.50–3.24	<0.01*			
Local Authority								
Wolverhampton	51	169	7.83	5.30–11.58	<0.01*	9.29	5.69–15.19	<0.01*
Place of birth								
UK-born	100	546	18.03	10.22–31.81	<0.01*	9.03	4.56–17.87	<0.01*
Ethnic group								
Black Caribbean	32	70	14.84	8.55–25.77	<0.01*	5.68	2.96–10.91	<0.01*
Black African	1	362	0.09	0.01–0.68	0.02	0.19	0.02–1.51	0.12
Indian Sub-Continent	33	1,102	1.00	1.00	Reference			
Other	6	105	1.57	0.60–4.10	0.36	1.11	0.37–3.37	0.85
White	49	366	4.47	2.79–7.15	<0.01*	1.75	0.95–3.22	0.07
Site of disease								
Pulmonary sputum smear positive	59	655	1.00	1.00	Reference			
Pulmonary sputum smear other	47	711	0.73	0.49–1.09	0.13	1.06	0.64–1.74	0.83
Extra pulmonary	18	685	0.29	0.17–0.50	<0.01*	0.62	0.33–1.18	0.15
Clinical history of TB								
Previous diagnosis of TB	10	85	0.60	0.30–1.20	0.15			
Treatment								
Patient admitted as in-patient	25	329	1.04	0.64–1.65	0.89			
Treatment outcome at 12 months								
Treatment completed	71	1,157	1.00	1.00	Reference			
Died	3	108	0.45	0.14–1.46	0.19			
Lost to follow up	4	72	0.91	0.32–2.55	0.85			
Still on treatment	2	32	1.02	0.24–4.34	0.98			
Treatment stopped	1	6	2.72	0.32–22.87	0.36			
Transferred out	0	18	-	-	-			
Not completed unknown	2	21	1.55	0.36–6.75	0.56			
Unknown	2	13	2.51	0.56–11.32	0.23			
Treatment outcome at 12 months								
Successful	73	1,189	0.89	0.47–1.66	0.71			
Drug Sensitivity Testing								
Resistance to any 1^st^ line drug	5	93	0.89	0.35–2.22	0.79			
MDR	1	15	1.10	0.14–8.43	0.92			

*P-values were considered as statistically significant if ≤0.05. Significant unadjusted values were included in the multi-variate model.

The wild-type (WT) consisted of tuberculosis patients infected with *M. tuberculosis* strains other than the Mercian strain.

### City-wide epidemiological investigation in Wolverhampton

The Mercian strain in Wolverhampton was significantly associated with white UK-born patients who presented with cavitations on chest X-ray and produced smear positive specimens (Table 2 and Table S2 for all epidemiological variables). Patients infected with the Mercian strain continued to experience weight loss at 8 weeks after starting anti-tubercular chemotherapy. This result was statistically significant (p<0.05). However, there was no significant difference between treatment completion rates after 12 months.

**Table 2:** Epidemiological data obtained from a city-wide investigation of patients with and without the Mercian strain in Wolverhampton, UK between 2003 and 2006.

			Unadjusted			Adjusted		
Variable	Mercian(n = 35)	WT(n = 47)	Odds Ratio	95% CI	p	Odds Ratio	95% CI	p
Patient gender								
Female	18	20	1.42	0.54–3.77	0.43			
Age group (years)								
0–14	1	1	1.15	0.01–92.99	1.00			
15–44	27	31	1.00	1.00	Reference			
45–64	4	10	0.46	0.10–1.85	0.22			
>65	3	5	0.69	0.10–3.95	0.72			
Ethnic group								
Indian Sub-Continent	11	23	1.00	1.00	Reference			
Black African	0	6	-	-	-			
Black Caribbean	9	6	3.06	0.75–13.46	0.07			
Other	1	5	0.44	0.01–4.71	0.65			
White	14	7	4.06	1.15–15.77	0.01*			
Country of birth								
UK-born	32	12	29.42	7.32–177.18	<0.01*	9.68	2.00–46.78	<0.01*
Epidemiological History								
Previous contact with TB case	24	11	6.94	2.42–21.50	<0.01*	3.40	0.92–12.59	0.07
Previous contact with Mercian strain	13	0	15.65	4.76–51.48	<0.01*			
Previous history of TB	9	3	4.97	1.11–31.13	0.01*			
Clinical co-factors								
Malignancy	1	0	10.41	0.20–547.59	0.43*			
Diabetes	2	7	0.35	0.03–2.01	0.29			
Social factors								
Excess alcohol use	16	4	8.78	2.42–41.01	<0.01*	6.26**	1.45–27.02	0.01
Cigarette smoking	6	3	2.99	0.58–19.96	0.12			
Cannabis use	11	2	10.02	1.96–100.33	<0.01*			
Unemployed	21	23	1.56	0.59–4.18	0.32			
Clinical presentation								
Cavitary disease	22	12	4.83	1.74–14.24	<0.01*	1.57	0.40–6.17	0.52
Pulmonary disease	31	34	2.93	0.79–13.64	0.07			
Sputum specimen	25	24	2.37	0.87–6.84	0.06			
Positive microscopy	22	17	2.94	1.10–8.19	0.02*			
Treatment								
Therapy non-adherence	7	3	3.61	0.75–23.42	0.06			
Weight loss after initiation of treatment	8	0	12.99	3.00–56.28	<0.01*			
Completed treatment within 12 months	26	36	0.64	0.17–2.29	0.43			
Drug Sensitivity Testing								
Resistance to any 1^st^ line drug	0	1	-	-	-			
MDR	0	0	-	-	-			

*P-values were considered as statistically significant if ≤0.05.

**OR for excess alcohol use and cannabis use combined.

Examination of the epidemiological factors revealed that cases with the Mercian strain were more likely to have a previous history of TB (9/35, 26%), and would have had significant previous contact with a case of TB (24/35, 69%), and in particular patients with the Mercian strain (13/35, 37%) (Table 2). Significant social factors detected were evidence of excess alcohol intake and cannabis use.

## Discussion

We describe here the identification of the most prevalent *M. tuberculosis* strain in the West Midlands, which we have termed the Mercian strain. Concordant MIRU-VNTR and RFLP data from six different geographical locations across the West Midlands indicated that this strain is present in 3 major cities. Regional, national, and global genotyping databases provided evidence that this strain was restricted to the West Midlands region in England. Regional data showed that this strain primarily infected UK-born, Black Caribbean patients less than 65 years old.

The regional and Wolverhampton epidemiological investigations presented in this report identified significant associations for the Mercian strain. However, they do not provide a full explanation of why the Mercian strain is more prevalent compared to other strains in the West Midlands. Drug and alcohol use were identified as significant social factors in Wolverhampton. Alcohol and drug use have been identified as significant associations in previously reported tuberculosis outbreaks particularly in low-incidence countries [19]–[22]. The cumulative number of cases and continuing presence of the Mercian strain does not follow a typical point-source outbreak pattern. The significant association with younger age suggests that cases caused by the Mercian strain have arisen as a result of recent transmission and not re-activation in older patients. A possible transmission scenario is that after the initial emergence of the Mercian strain there have been several independent clusters of transmission each with their own common social link. This has resulted in a large, complex social network where transmission persists and the complete transmission scenario is yet to be fully elucidated.

Both epidemiological investigations presented in this report were retrospective and did not involve direct patient interviews. The Mercian strain continues to be identified in the West Midlands which means that enhanced epidemiological knowledge could be obtained by prospectively investigating social links as each new patient is diagnosed. Investigation of potential factors which may cause a delay in diagnosis should be investigated as well. The data presented by us identified the infected patient population and also important common social factors. The exact interaction of patient population and social factors should be investigated further to identify and fully understand any confounding factors.

It must be noted that the Wolverhampton epidemiological investigation applied a detailed questionnaire that was only used in this location. Of the three major cities in the West Midlands, Wolverhampton had the highest proportion of the Mercian strain (21%). Patients with the Mercian strain in Birmingham and Coventry might differ in their use of drugs and alcohol. The results from the Wolverhampton and region-wide analysis do not concord exactly as different ethnic population groups were identified as at highest risk: the White population in Wolverhampton but the Black Caribbean group across the West Midlands.

Detection of this strain was only possible with the commencement of universal prospective typing of all *M. tuberculosis* isolates in the West and East Midlands. Only with universal prospective DNA fingerprinting was the full extent of the Mercian strain in the West Midlands fully characterized. Since the Mercian strain is not a drug-resistant strain without associated phenotypic properties that could differentiate it from other *M. tuberculosis* complex strains, it would only have been possible to detect this strain by universal prospective typing. The patient population in which the Mercian strain has been identified is different to the UK-wide situation for TB as the majority of patients diagnosed each year in the UK are not born in the UK and originate from the Indian Sub-Continent [9].

The 156 individual patients detected between 2004 and 2008, make the Mercian strain one of the largest known community-based clusters in the world. Previous major prevalent strains have been identified in New York [23], [24], Rotterdam [25], North London [3], and Rio de Janeiro [26].

The most prevalent strain detected in the UK was an isoniazid resistant strain in North London that was previously reported in 70 patients [3], with a current total of over 300 cases caused by this strain (Ibrahim Abubakar, Consultant Epidemiologist & TB Section Head, Respiratory Diseases Department - Tuberculosis Section, Health Protection Agency, personal communication). Isoniazid resistance acted as a very useful marker for detection of the strain. It was noted that without the drug resistance marker only prospective typing of all isolates would have detected this large, complex outbreak. This strain was predominantly found in young White or Black Caribbean UK-born adults with drug misuse as a common epidemiological factor [3]. It is possible that patients in this population group take longer to present clinically as TB may not be suspected when initial symptoms develop or they might not seek medical help soon after onset. Both factors aid strain transmission and disease progression.

A strain was identified in 93/314 (30%) patients in Rio de Janeiro that uniquely lacked a major region of genomic DNA (>26.3 kb) which contained 10 genes including two potentially immunogenic PPE genes [26], [27]. The RD^Rio^ strain was associated with a higher frequency of cavitary pulmonary disease [28]. The major deletion identified in the RD^Rio^ strain has been hypothesized as having a major impact on the virulence properties of the RD^Rio^ strain. As the genomic content of the Mercian strain has not been characterized, further work should determine whether such a deletion or other similar major genomic variation has altered the virulence of this strain leading to multiple transmission events in and between three cities in the West Midlands.

Retrospective epidemiological studies have identified the earliest isolate of the Mercian strain from 1995 in an archive strain collection. This isolate was part of a cluster of 11 isoniazid resistant strains identified between 1995 and 2000 which was reported previously before the full regional extent of the Mercian strain was known [2]. We have typed very few archived *M. tuberculosis* strains from 1995 so the full extent of drug sensitive and drug resistant Mercian strains 15 years ago has not yet been assessed. The cluster of isoniazid resistant Mercian strains was present in one specific location. From 1995–2000, there was no other investigated instance of increased isoniazid resistance in the rest of the West Midlands caused by the Mercian strain.

As the Mercian strain has been present since prospective DNA fingerprinting was commenced in 2004 with a median of 30 isolates per year (range 27–37) and has represented a consistent proportion of strains (Figure 1), it is likely that the Mercian strain first emerged in the West Midlands well before 2004.

Universal prospective DNA fingerprinting is an essential part of many countries TB control programs as it has been used to estimate transmission in specific populations groups. The Netherlands have undertaken universal prospective DNA fingerprinting of all *M. tuberculosis* isolates since 1993 [29]. This enabled the identification of transmission after migration in patients recently arrived in the Netherlands as infections in 30% to 40% of Turkish, Moroccan, and Somali patients could be attributed to recent transmission [29].

Secondly, it has also been shown in the Netherlands that combining data obtained from nationwide tuberculosis contact investigation and DNA fingerprinting surveillance greatly increased the number of defined epidemiological links. In 2,206 clustered cases, DNA fingerprinting increased the number of epidemiologic links from 462 before DNA fingerprinting data was known to 1,002 epidemiologic established links after cluster investigation involving a combination of molecular and epidemiological data. DNA fingerprinting did not increase the number of patients identified as contacts but cluster monitoring did enable the identification of transmission events not detected by contact investigations, the development and evaluation of focused interventions and evaluation of regional tuberculosis eradication programs [30]. A study in Maryland showed that cluster investigation including DNA fingerprinting analysis identified 43/113 (38%) of all detected patient links. [31] .

A more recent study showed that DNA fingerprinting data could be used to prospectively identify rapidly expanding clusters before expansion actually occurred based on the properties of the first two patients in a cluster. If the first two patients in a cluster were identified within 3 months of each other, one or both were <35 years old, and both patients resided in an urban area and originated from Sub-Saharan Africa, there was a more than 5 times increased probability that this strain in an initial cluster of two paired patients would be identified in 5 or more patients within 2 years [32].

We describe here the identification of the most prevalent *M. tuberculosis* strain in the West Midlands region of the UK, with 156 isolates in a 5 year period between 2004 and 2008. The Mercian strain has been significantly associated with UK-born patients, appears to be geographically restricted to the West Midlands region in the UK with evidence of ongoing transmission.

## Supporting Information

Table S1 Univariate and multi-variate analysis of sociodemographic, clinical, and bacteriological data for patients with the Mercian strain (n = 124) and all other patients with strain typing data (n = 2,066) in the West Midlands from 2004–2008.
(DOCX)

Table S2 Epidemiological data obtained from a city-wide investigation of patients with and without the Mercian strain in Wolverhampton, UK.
(DOCX)
