# Supplementary material for: A Geographically-Restricted but Prevalent Mycobacterium tuberculosis Strain Identified in the West Midlands Region of the UK between 1995 and 2008
Source: PLoS One. 2011 Mar 25;6(3):e17930. doi: 10.1371/journal.pone.0017930 (PMC3064665; doi:10.1371/journal.pone.0017930)
Supplement: Table S1 — Univariate and multi-variate analysis of sociodemographic, clinical, and bacteriological data for patients with the Mercian strain (n = 124) and all other patients with strain typing data (n = 2,066) in the West Midlands from 2004–2008. (DOCX) [file pone.0017930.s001.docx]

|  | **No. patients** | | **Unadjusted** | | |  | **Adjusted** | | |
| --- | --- | --- | --- | --- | --- | --- | --- | --- | --- |
| **Variable** | **Mercian**  **(n=124)** | **WT**  **(n=2,066)** | **Odds Ratio** | **95% CI** | **P** |  | **Odds Ratio** | **95% CI** | **p** |
| Gender |  |  |  |  |  |  |  |  |  |
| Male | 77 | 1,118 | 1.38 | 0.95-2.01 | 0.09 |  | 1.03 | 0.65-1.62 | 0.91 |
| Female | 47 | 944 | 1.00 | 1.00 | Reference |  |  |  |  |
| Age group (years) |  |  |  |  |  |  |  |  |  |
| 0-14 | 4 | 45 | 1.27 | 0.45-3.59 | 0.69 |  | 0.35 | 0.07-1.63 | 0.18 |
| 15-44 | 92 | 1310 | 1.00 | 1.00 | Reference |  |  |  |  |
| 45-64 | 23 | 379 | 0.86 | 0.54-1.38 | 0.543 |  | 0.77 | 0.42-1.41 | 0.40 |
| >65 | 5 | 330 | 0.22 | 0.09-0.53 | <0.01* |  | 0.25 | 0.09-0.67 | <0.01* |
| HPU location in the West Midlands |  |  |  |  |  |  |  |  |  |
| East | 47 | 1,126 | 1.00 | 1.00 | Reference |  |  |  |  |
| North | 10 | 206 | 1.16 | 0.58-2.34 | 0.67 |  |  |  |  |
| West | 67 | 727 | 2.21 | 1.50-3.24 | <0.01* |  |  |  |  |
| Local Authority |  |  |  |  |  |  |  |  |  |
| Wolverhampton | 51 | 169 | 7.83 | 5.30-11.58 | <0.01* |  | 9.29 | 5.69-15.19 | <0.01* |
| 32 other Local Authorities | 73 | 1,895 | 1.00 | 1.00 | Reference |  |  |  |  |
| Place of birth |  |  |  |  |  |  |  |  |  |
| UK-born | 100 | 546 | 18.03 | 10.22-31.81 | <0.01* |  | 9.03 | 4.56-17.87 | <0.01* |
| Non-UK-born | 14 | 1,378 | 1.00 | 1.00 | Reference |  |  |  |  |
| Ethnic group |  |  |  |  |  |  |  |  |  |
| Black Caribbean | 32 | 70 | 14.84 | 8.55-25.77 | <0.01* |  | 5.68 | 2.96-10.91 | <0.01* |
| Black African | 1 | 362 | 0.09 | 0.01-0.68 | 0.02 |  | 0.19 | 0.02-1.51 | 0.12 |
| Indian Sub-Continent | 33 | 1,102 | 1.00 | 1.00 | Reference |  |  |  |  |
| Other | 6 | 105 | 1.57 | 0.60-4.10 | 0.36 |  | 1.11 | 0.37-3.37 | 0.85 |
| White | 49 | 366 | 4.47 | 2.79-7.15 | <0.01* |  | 1.75 | 0.95-3.22 | 0.07 |
| Site of disease |  |  |  |  |  |  |  |  |  |
| Pulmonary sputum smear positive | 59 | 655 | 1.00 | 1.00 | Reference |  |  |  |  |
| Pulmonary sputum smear other | 47 | 711 | 0.73 | 0.49-1.09 | 0.13 |  | 1.06 | 0.64-1.74 | 0.83 |
| Extra pulmonary | 18 | 685 | 0.29 | 0.17-0.50 | <0.01* |  | 0.62 | 0.33-1.18 | 0.15 |
| Clinical history of TB |  |  |  |  |  |  |  |  |  |
| Previous diagnosis of TB | 10 | 85 | 0.60 | 0.30-1.20 | 0.15 |  |  |  |  |
| No previous diagnosis of TB | 82 | 1,164 | 1.00 | 1.00 | Reference |  |  |  |  |
| Treatment |  |  |  |  |  |  |  |  |  |
| Patient admitted as in-patient | 25 | 329 | 1.04 | 0.64-1.65 | 0.89 |  |  |  |  |
| Patient admitted as out-patient | 58 | 737 | 1.00 | 1.00 | Reference |  |  |  |  |
| Treatment outcome at 12 months |  |  |  |  |  |  |  |  |  |
| Treatment completed | 71 | 1,157 | 1.00 | 1.00 | Reference |  |  |  |  |
| Died | 3 | 108 | 0.45 | 0.14-1.46 | 0.19 |  |  |  |  |
| Lost to follow up | 4 | 72 | 0.91 | 0.32-2.55 | 0.85 |  |  |  |  |
| Still on treatment | 2 | 32 | 1.02 | 0.24-4.34 | 0.98 |  |  |  |  |
| Treatment stopped | 1 | 6 | 2.72 | 0.32-22.87 | 0.36 |  |  |  |  |
| Transferred out | 0 | 18 | - | - | - |  |  |  |  |
| Not completed unknown | 2 | 21 | 1.55 | 0.36-6.75 | 0.56 |  |  |  |  |
| Unknown | 2 | 13 | 2.51 | 0.56-11.32 | 0.23 |  |  |  |  |
| Treatment outcome at 12 months |  |  |  |  |  |  |  |  |  |
| Successful | 73 | 1,189 | 0.89 | 0.47-1.66 | 0.71 |  |  |  |  |
| Not successful | 12 | 220 | 1.00 | 1.00 | Reference |  |  |  |  |
| Drug Sensitivity Testing |  |  |  |  |  |  |  |  |  |
| Resistance to any 1^st^ line drug | 5 | 93 | 0.89 | 0.35-2.22 | 0.79 |  |  |  |  |
| No resistance to any 1^st^ line drug | 119 | 1,959 | 1.00 | 1.00 | Reference |  |  |  |  |
| MDR | 1 | 15 | 1.10 | 0.14-8.43 | 0.92 |  |  |  |  |
| Not MDR | 123 | 2,037 | 1.00 | 1.00 | Reference |  |  |  |  |

*P-values were considered as statistically significant if ≤0.05. Significant unadjusted values were included in the multi-variate model.
