# Supplementary material for: A Geographically-Restricted but Prevalent Mycobacterium tuberculosis Strain Identified in the West Midlands Region of the UK between 1995 and 2008
Source: PLoS One. 2011 Mar 25;6(3):e17930. doi: 10.1371/journal.pone.0017930 (PMC3064665; doi:10.1371/journal.pone.0017930)
Supplement: Table S2 — Epidemiological data obtained from a city-wide investigation of patients with and without the Mercian strain in Wolverhampton, UK. (DOCX) [file pone.0017930.s002.docx]

|  |  |  | **Unadjusted** | | |  | **Adjusted** | | |
| --- | --- | --- | --- | --- | --- | --- | --- | --- | --- |
| **Variable** | **Mercian**  **(n=35)** | **WT**  **(n=47)** | **Odds Ratio** | **95% CI** | **p** |  | **Odds Ratio** | **95% CI** | **p** |
| Patient gender |  |  |  |  |  |  |  |  |  |
| Female | 18 | 20 | 1.42 | 0.54-3.77 | 0.43 |  |  |  |  |
| Male | 17 | 27 | 1.00 | 1.00 | Reference |  |  |  |  |
| Age group (years) |  |  |  |  |  |  |  |  |  |
| 0-14 | 1 | 1 | 1.15 | 0.01-92.99 | 1.00 |  |  |  |  |
| 15-44 | 27 | 31 | 1.00 | 1.00 | Reference |  |  |  |  |
| 45-64 | 4 | 10 | 0.46 | 0.10-1.85 | 0.22 |  |  |  |  |
| >65 | 3 | 5 | 0.69 | 0.10-3.95 | 0.72 |  |  |  |  |
| Ethnic group |  |  |  |  |  |  |  |  |  |
| Indian Sub-Continent | 11 | 23 | 1.00 | 1.00 | Reference |  |  |  |  |
| Black African | 0 | 6 | - | - | - |  |  |  |  |
| Black Caribbean | 9 | 6 | 3.06 | 0.75-13.46 | 0.07 |  |  |  |  |
| Other | 1 | 5 | 0.44 | 0.01-4.71 | 0.65 |  |  |  |  |
| White | 14 | 7 | 4.06 | 1.15-15.77 | 0.01* |  |  |  |  |
| Country of birth |  |  |  |  |  |  |  |  |  |
| UK-born | 32 | 12 | 29.42 | 7.32-177.18 | <0.01* |  | 9.68 | 2.00-46.78 | <0.01* |
| Non-UK-born | 3 | 35 | 1.00 | 1.00 | Reference |  |  |  |  |
| Epidemiological History |  |  |  |  |  |  |  |  |  |
| Previous contact  with TB case | 24 | 11 | 6.94 | 2.42-21.50 | <0.01* |  | 3.40 | 0.92-12.59 | 0.07 |
| No previous contact  with TB case | 11 | 36 | 1.00 | 1.00 | Reference |  |  |  |  |
| Previous contact  with Mercian strain | 13 | 0 | 15.65 | 4.76-51.48 | <0.01* |  |  |  |  |
| No previous contact  with Mercian strain | 13 | 46 | 1.00 | 1.00 | Reference |  |  |  |  |
| Previous history of TB | 9 | 3 | 4.97 | 1.11-31.13 | 0.01* |  |  |  |  |
| No previous history of TB | 26 | 44 | 1.00 | 1.00 | Reference |  |  |  |  |
| Clinical co-factors |  |  |  |  |  |  |  |  |  |
| Malignancy | 1 | 0 | 10.41 | 0.20-547.59 | 0.43* |  |  |  |  |
| No evidence of malignancy | 34 | 47 | 1.00 | 1.00 | Reference |  |  |  |  |
| Diabetes | 2 | 7 | 0.35 | 0.03-2.01 | 0.29 |  |  |  |  |
| No evidence of diabetes | 33 | 40 | 1.00 | 1.00 | Reference |  |  |  |  |
| Social factors |  |  |  |  |  |  |  |  |  |
| Excess alcohol use | 16 | 4 | 8.78 | 2.42-41.01 | <0.01* |  | 6.26** | 1.45-27.02 | 0.01 |
| No evidence of  excess alcohol use | 19 | 43 | 1.00 | 1.00 | Reference |  |  |  |  |
| Cigarette smoking | 6 | 3 | 2.99 | 0.58-19.96 | 0.12 |  |  |  |  |
| Non-smoker | 29 | 44 | 1.00 | 1.00 | Reference |  |  |  |  |
| Cannabis use | 11 | 2 | 10.02 | 1.96-100.33 | <0.01* |  |  |  |  |
| No evidence of  cannabis use | 24 | 45 | 1.00 | 1.00 | Reference |  |  |  |  |
| Employed | 14 | 24 | 1.00 | 1.00 | Reference |  |  |  |  |
| Unemployed | 21 | 23 | 1.56 | 0.59-4.18 | 0.32 |  |  |  |  |
| Clinical presentation |  |  |  |  |  |  |  |  |  |
| Cavitary disease | 22 | 12 | 4.83 | 1.74-14.24 | <0.01* |  | 1.57 | 0.40-6.17 | 0.52 |
| Non-cavitary disease | 13 | 35 | 1.00 | 1.00 | Reference |  |  |  |  |
| Pulmonary disease | 31 | 34 | 2.93 | 0.79-13.64 | 0.07 |  |  |  |  |
| Non-pulmonary disease | 4 | 13 | 1.00 | 1.00 | Reference |  |  |  |  |
| Sputum specimen | 25 | 24 | 2.37 | 0.87-6.84 | 0.06 |  |  |  |  |
| No sputum specimen | 10 | 23 | 1.00 | 1.00 | Reference |  |  |  |  |
| Positive microscopy | 22 | 17 | 2.94 | 1.10-8.19 | 0.02* |  |  |  |  |
| Negative microscopy | 13 | 30 | 1.00 | 1.00 | Reference |  |  |  |  |
| Treatment |  |  |  |  |  |  |  |  |  |
| Therapy adherence | 28 | 44 | 1.00 | 1.00 | Reference |  |  |  |  |
| Therapy non-adherence | 7 | 3 | 3.61 | 0.75-23.42 | 0.06 |  |  |  |  |
| No weight loss after  initiation of treatment | 27 | 47 | 1.00 | 1.00 | Reference |  |  |  |  |
| Weight loss after  initiation of treatment | 8 | 0 | 12.99 | 3.00-56.28 | <0.01* |  |  |  |  |
| Completed treatment  within 12 months | 26 | 36 | 0.64 | 0.17-2.29 | 0.43 |  |  |  |  |
| Did not complete  treatment within 12 months | 8 | 7 | 1.00 | 1.00 | Reference |  |  |  |  |
| Drug Sensitivity Testing |  |  |  |  |  |  |  |  |  |
| Resistance to any 1^st^ line drug | 0 | 1 | - | - | - |  |  |  |  |
| No resistance to any 1^st^ line drug | 35 | 46 | 1.00 | 1.00 | Reference |  |  |  |  |
| MDR | 0 | 0 | - | - | - |  |  |  |  |
| Not MDR | 35 | 47 | 1.00 | 1.00 | Reference |  |  |  |  |

*P-values were considered as statistically significant if ≤0.05.

**OR for excess alcohol use and cannabis use combined.
